# From dyspnea to diagnosis, unmasking undifferentiated cardiac sarcoma: a case report

**DOI:** 10.1186/s43044-024-00520-3

**Published:** 2024-07-06

**Authors:** Mauricio Guerra-Raygada, Alee Jonhson Saavedra-Sanchez, Diego Hidalgo-Avendaño, Milagros F. Bermudez-Pelaez, David Guevara-Lazo, Natalia Nombera-Aznaran

**Affiliations:** 1 Hospital Nacional Arzobispo Loayza, Lima, Peru; 2 Alberto Hurtado Faculty of Human Medicine, Universidad Peruana Cayetano Heredia, Jirón Nicolas Poussin 101, San Borja, Lima, Peru; 3 Department of Medical Specialties, Cardiology and Coronary Care Service, Lima, Peru

**Keywords:** Heart neoplasm, Sarcoma, Heart failure

## Abstract

**Background:**

Sarcomas are the most common type of cardiac malignancy, but they are extremely rare. Within this group, angiosarcomas have the highest frequency, followed by undifferentiated sarcomas. This type of tumor has a poor prognosis and a high recurrence rate. Information about these tumors is limited, relying mainly on case reports and autopsy series. The purpose of this case report is to detail the multifaceted approach to diagnosing and managing an undifferentiated cardiac sarcoma and contribute to the literature.

**Case presentation:**

A 28-year-old man presented with dyspnea and chest pain, which had developed progressively over several weeks. Physical examination revealed low blood pressure, elevated heart rate, and diminished heart sounds. Imaging, including a CT scan, identified a hypodense mass in the right ventricle. Further evaluation through echocardiograms and contrast angiotomography confirmed a mass causing right ventricular obstruction. Part of the tumor was surgically removed and diagnosed  as cardiac sarcoma.  Histopathological analysis of the mass showed an undifferentiated cardiac sarcoma.

**Conclusion:**

This case underscores the significance of including cardiac tumors as a potential cause when diagnosing cardiac masses. It also demonstrates the poor prognosis and tendency for recurrence, while revealing the absence of established management guidelines.

**Supplementary Information:**

The online version contains supplementary material available at 10.1186/s43044-024-00520-3.

## Background

Cardiac masses are rare and include benign, malignant (both primary and secondary), and tumor-like conditions (e.g., thrombi, vegetations, or pericardial cysts) [1, 2]. Sarcomas are the most common type of malignant cardiac neoplasms, though they are exceedingly rare. Among sarcomas, angiosarcomas, followed by leiomyosarcomas, are the most common differentiated sarcomas, followed by undifferentiated sarcomas. These neoplasms lack a predominant histological pattern, are invasive, and tend to be located in the left atrium [1]. Despite treatment with surgery and palliative chemotherapy, the prognosis remains poor.

**Fig. 1:**
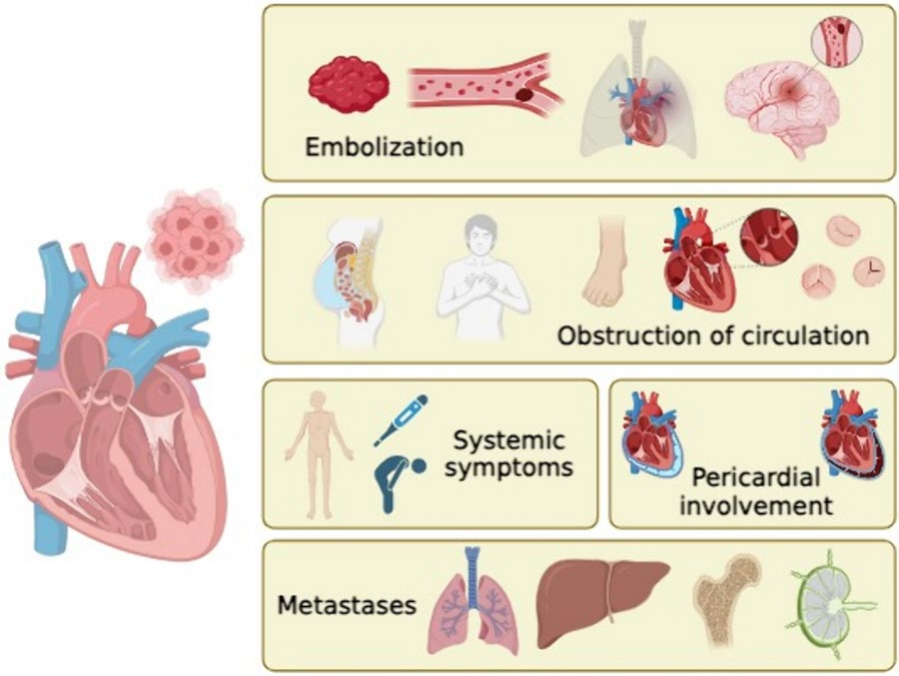
Symptoms and complications of cardiac masses

Clinical presentation can be either asymptomatic or symptomatic. Cardiac manifestations result from the physical space occupied by the mass within the cardiac cavity. Tumors located in the right ventricle, such as in our patient, often interfere with blood filling and/or ejection from the ventricle, resulting in right-sided heart failure (Fig. 1) [3]. Clinical evaluation begins with the use of cardiac imaging to determine morphology, size, location, mobility, and extension to the pericardium or adjacent structures. Additionally, imaging aids in assessing hemodynamic status and designing the surgical plan [1, 3].

Cardiac sarcomas are exceedingly rare with a poor prognosis, and knowledge about these tumors is limited, relying on case reports and autopsy series. The aim of this case report is to present the management and diagnosis of an undifferentiated cardiac sarcoma, understand the role of histopathologic diagnosis in the prognosis and recurrence of cardiac tumor diseases, and contribute to the existing literature.

## Case presentation

A 28-year-old male was admitted to the emergency department with symptoms of dyspnea and chest pain. The dyspnea had begun four weeks prior, initially occurring during exertion and eventually affecting daily activities. Pleuritic chest pain appeared two weeks later. The patient was previously diagnosed with active pulmonary tuberculosis three years ago and underwent successful treatment for six months with standard tuberculosis medications. He had no prior history of similar episodes and had a non-significant medical history.

Upon examination, the patient was conscious but in poor general condition, with a blood pressure of 90/60 mmHg, a heart rate of 114 bpm, a respiratory rate of 27 rpm, and oxygen saturation of 84% without supplemental oxygen. Cardiac examination revealed a regular rhythm with a diminished second sound and a grade III holosystolic murmur heard best over the left upper sternal border. Peripheral edema and jugular venous pulsation were absent. The pulmonary examination showed crackles, which were best heard in both lung bases. Other findings were unremarkable.

**Fig. 2:**
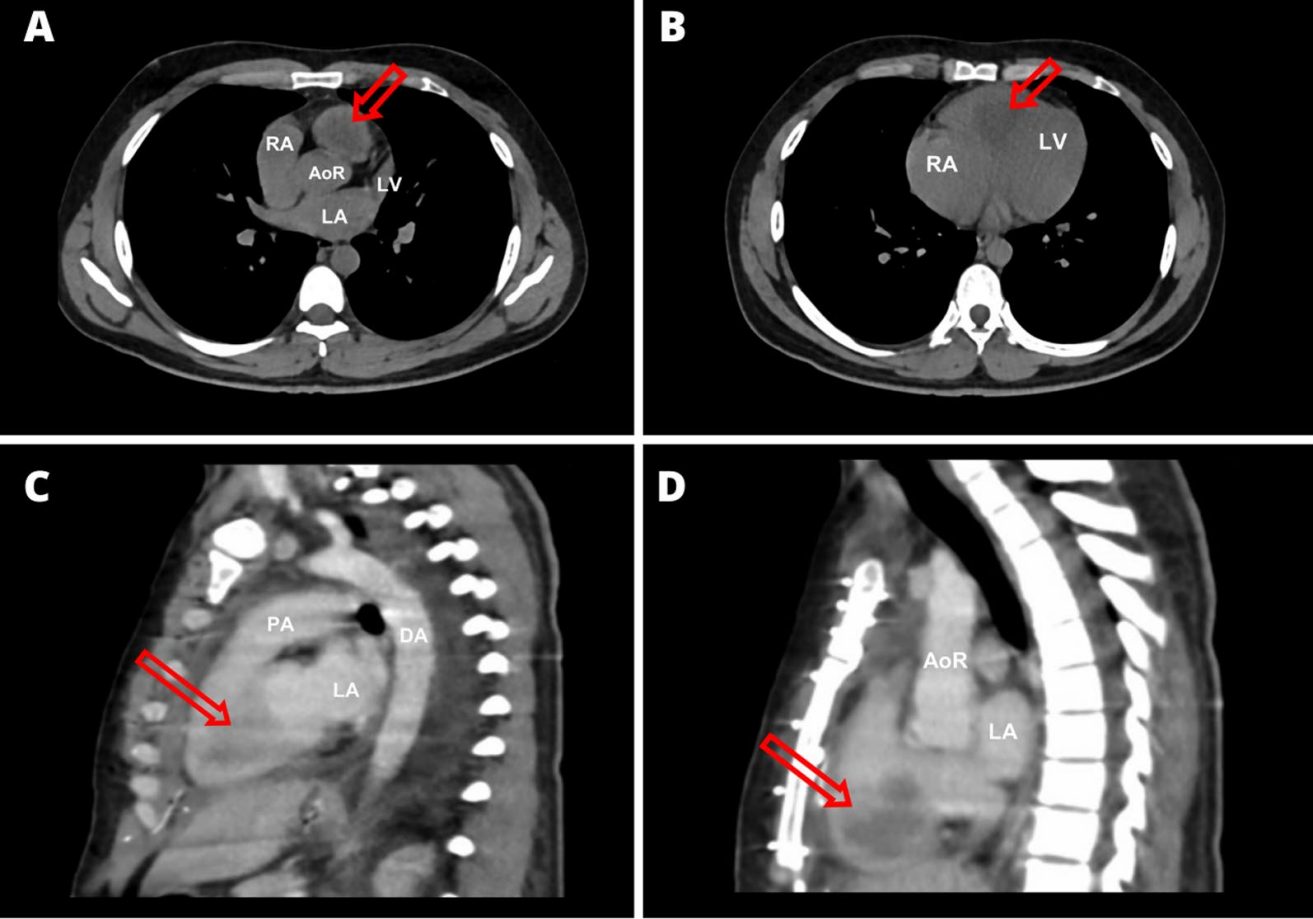
Axial CT scans without contrast **A** and **B** depict a mass occupying the right ventricular outflow tract (arrow). Sagittal CT scans with contrast **C** and **D** reveal a mass in the right ventricle (arrow). AoR: root of the aorta, RA: right atrium, LA: left atrium, LV: left ventricle, DA: descendent aorta, and PA: pulmonary artery

A SARS-CoV-2 antigen test was negative. Initial laboratory tests revealed an elevated white blood cell count of 14.9 × 10^9^/L, metabolic acidosis, and hyponatremia. An electrocardiogram showed sinus rhythm, complete right bundle branch block, and right ventricular systolic overload. A computed tomography (CT) scan revealed fibroatelectatic tracts, traction bronchiectasis, pleural thickening, and a hypodense mass in the right ventricle attached to the interventricular septum (Fig. 2).

**Fig. 3:**
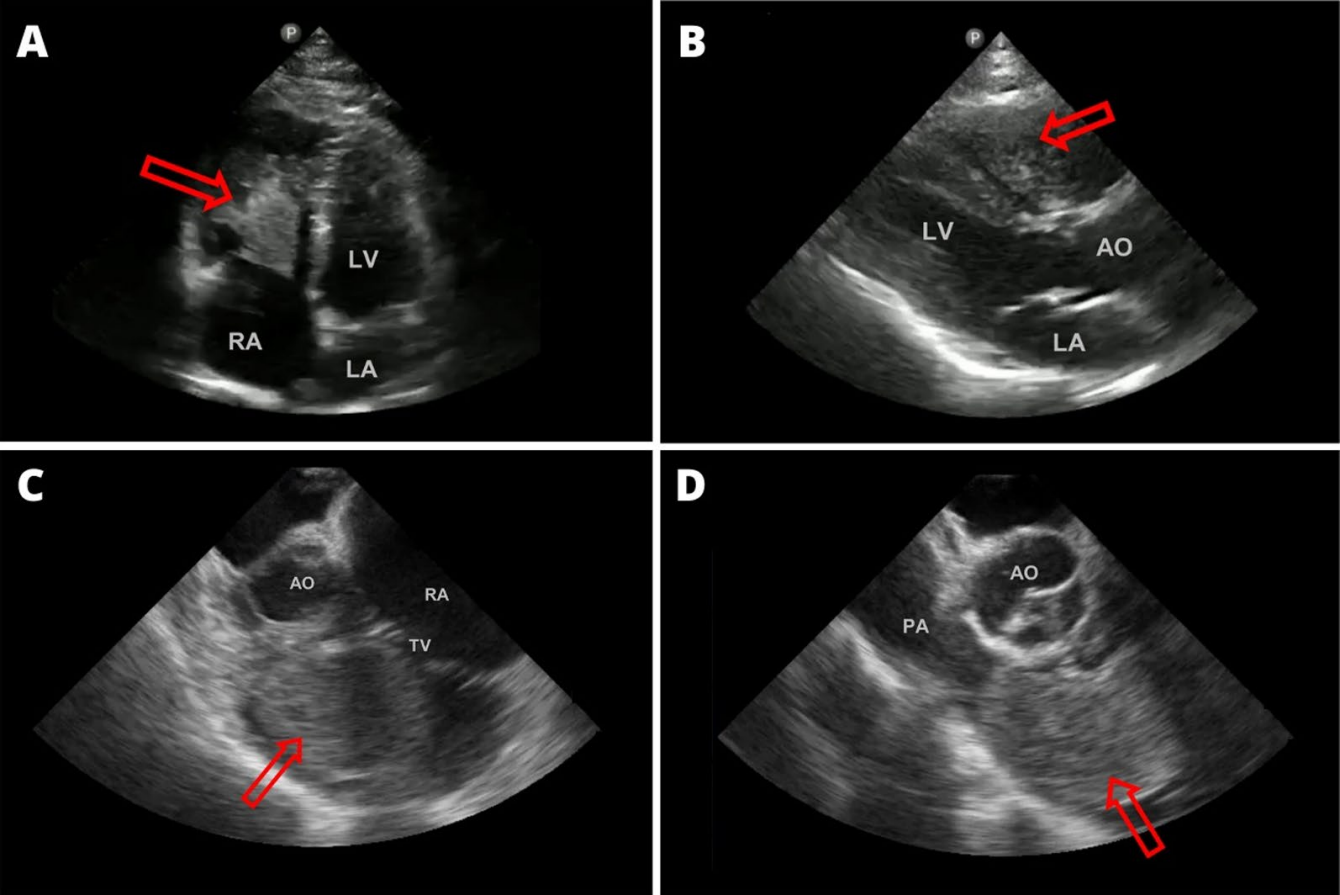
**A** Four-chamber apical view, transthoracic echocardiography, showing a heterogeneous, pedunculated mass (arrow) protruding toward the septal leaflet of the tricuspid valve. **B** Long-axis parasternal view, transthoracic echocardiography, displaying a heterogeneous mass (arrow) attached to the interventricular septum. **C** Mid-esophageal projection, displaying a solid tumor mass protruding toward the tricuspid valve. **D** Mid-esophageal projection at the level of the great vessels, displaying the tumor mass in the right ventricular outflow tract. RA: right atrium, LA: left atrium, LV: left ventricle, AO: aorta, TV: tricuspid valve, and PA: pulmonary artery

The suspected diagnosis of pulmonary thromboembolism was ruled out after an angiotomography. Instead, it revealed a pedunculated intracardiac mass in the right ventricle attached to the interventricular septum, leading to the conclusion that a defect in ventricular filling was causing the patient’s symptoms (Fig. 2C and D). Transthoracic and transesophageal echocardiograms were performed to further characterize the mass (Fig. 3). The transthoracic echocardiogram showed an image with heterogeneous echogenicity in the right ventricle. The transesophageal echocardiogram revealed a heterogeneous mass with regular borders (36 × 46 mm) attached to the septal wall of the basal interventricular septum of the right ventricle, causing dynamic obstruction of the right ventricular outflow tract. Furthermore, mild tricuspid regurgitation and a dilated right ventricle were observed. Following this, a thoracoabdominal pelvic CT scan with contrast was performed to investigate the presence of a potential primary tumor or systemic metastases, which revealed no other abnormal findings.

**Fig. 4:**
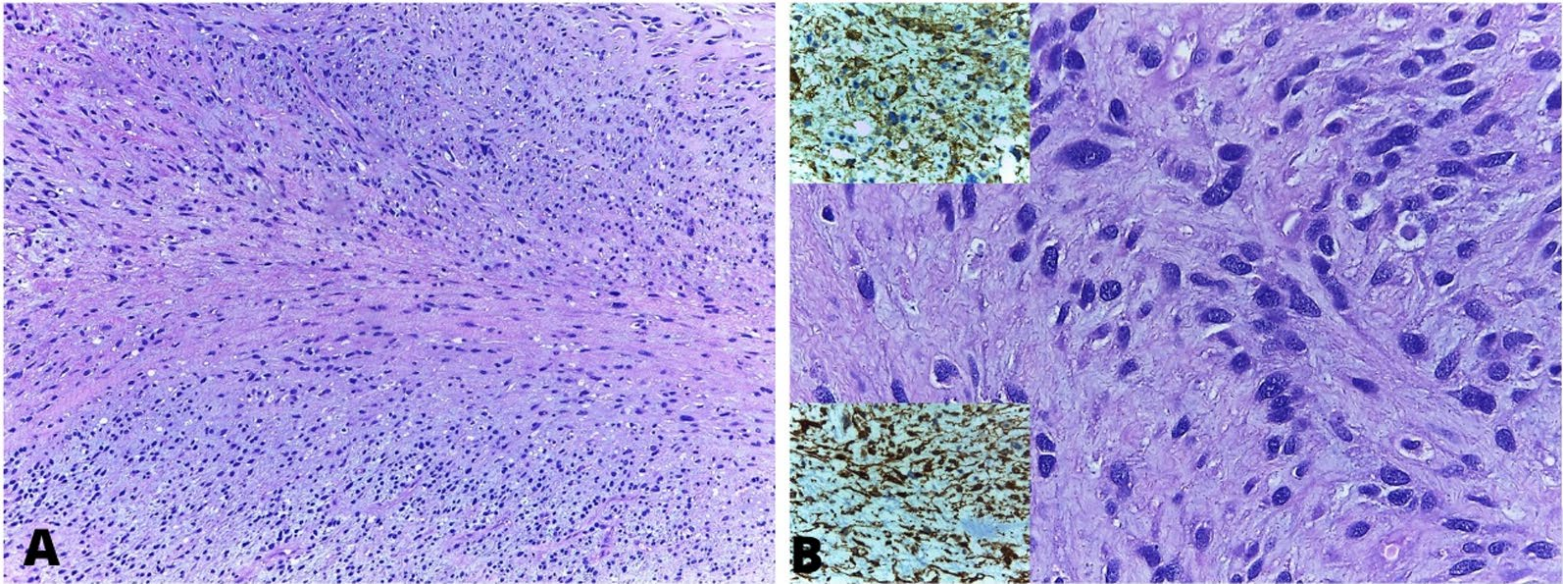
**A.** Low magnification (4×) view displaying cell proliferation arranged in fascicles on a myxoid-appearing, basophilic stroma (HE). **B.** High magnification (40×) revealing neoplastic cells with elongated, hyperchromatic nuclei and moderate nuclear pleomorphism. Top image shows positive actin staining, while the bottom image shows positive desmin staining

The surgical resection plan aimed to remove the tumor; however, its anatomic features prevented complete removal, leaving a portion attached to the base of the right ventricle’s inferior and septal wall. After the removal of the mass, mediastinal and pericardial drainage was performed, along with the closure of both cavities. The mass was solid, fibrous, and pearly white, with a base of approximately 4 cm and an extension of 10 cm toward the right ventricular outflow tract. Tissue analysis confirmed a diagnosis of cardiac sarcoma (Fig. 4).

After surgery, the patient was admitted to the coronary intensive care unit (ICU). He developed a hospital-acquired pneumonia associated with mechanical ventilation and was treated with broad-spectrum antibiotics, which improved his condition. An echocardiogram before discharge showed a residual solid mass attached to the interventricular septum. The right ventricular systolic function was reduced but the left ventricular function was preserved. The patient was transferred to a cancer center to receive chemotherapy and radiotherapy. Amid ongoing evaluation, cancer treatment had not yet started when his health declined, evidenced by tachypnea and dyspnea at rest, ultimately leading to readmission to the hospital 20 days after discharge. Upon arrival, he was hemodynamically unstable, with oxygen saturation ranging from 70 to 80%, a heart rate of 120–130 bpm, and hypotension. He was promptly placed on mechanical ventilation in the ICU. A recurrence of the cardiac mass was suspected, leading to further obstruction of the outflow tract of the right ventricle. Despite medical intervention, his condition continued to deteriorate, and after one week of admission, he died.

## Discussion

Tumors of the heart can be classified according to “The 2021 WHO Classification of Tumors of the Heart.” They are separated into benign tumors, tumors of uncertain biologic behavior, germ cell tumors, and malignant tumors (Table 1). Most of the tumors are benign involving myxomas, rhabdomyomas, papillary fibroelastoma, hemangiomas, etc. Malignant tumors include sarcomas, metastases, hematolymphoid tumors, etc. [2]. Primary malignant cardiac tumors are extremely rare, an analysis from the Surveillance, Epidemiology, and End Results database from the National Cancer Institute identified 694 cases from 1973 to 2015 out of a total of 7 384 580 cancer cases registered, accounting for only 0.009% of all cancers [4].

**Table 1:** International Classification of Heart Tumors According to the 2021 World Health Organization

Benign tumors	Malignant tumors
Papillary fibroelastoma Myxoma Fibroma Rhabdomyoma Adult cellular rhabdomyoma Lipoma Lipomatous hypertrophy of the atrial septum Lipomatous hamartoma of atrioventricular valve Hamartoma of mature cardiac myocytes Mesenchymal cardiac hamartoma Hemangioma Venous hemangioma Capillary hemangioma Arteriovenous hemangioma Cavernous hemangioma Conduction system hamartoma Cystic tumor of atrioventricular node	Angiosarcoma Leiomyosarcoma Pleomorphic sarcoma Neoplasm, metastatic Diffuse large B cell lymphoma Fibrin-associated diffuse large B cell lymphoma

Cardiac tumors can be asymptomatic or found incidentally during the evaluation of another condition. However, when they do manifest clinical features, the presentation depends on the location and size of the tumor. In this context, the clinical presentation is often determined by the mass effect of the tumor, embolic phenomena, and direct invasion of the myocardium or lungs. Dyspnea is the most common symptom, followed by chest pain, heart failure, palpitations, syncope, and emboli [3, 5]. Our patient experienced symptoms of right heart failure due to obstruction of the right ventricular outflow tract and an audible murmur due to the tumor protruding through the valves. At the time of diagnosis, approximately 66% to 89% of patients with primary cardiac angiosarcoma have metastasis, commonly in the lung and bone and occasionally in the liver and brain [6, 7].

The use of cardiac imaging is essential for characterizing the mass. Echocardiography is the initial test used to assess hemodynamic compromise, size, location, mobility, and extent of the tumor. Although cardiac magnetic resonance offers comprehensive evaluation and better correlation with histopathology, it is not available at our hospital. CT scan and magnetic resonance imaging are valuable for tumor staging, detecting metastases, and planning surgical procedures [1]. The diagnosis is confirmed through postoperative histopathological analysis.

Surgical excision of primary cardiac sarcoma is the only treatment shown to increase survival [8]. In our patient, partial resection was performed due to the tumor’s location, aimed at preserving cardiac function. Postoperative surveillance is vital because sarcoma recurs in 50% of cases with complete resection [8]. In addition, patients with negative surgical margins have a longer median overall survival than those with positive margins. A retrospective case series involving 32 patients who underwent surgical resection of malignant cardiac tumors showed a median survival time of three years, with estimated survival rates at 6 months and 1, 5, and 10 years of 90%, 73%, 31%, and 17%, respectively [8]. Our patient was scheduled to receive chemotherapy and radiotherapy, but their roles in managing cardiac sarcomas are not fully elucidated. The use of radiotherapy is limited due to the heart’s poor tolerance, although it is indicated for managing positive margins after resection, palliating localized aggressive disease, or addressing recurrence [9]. However, in another study of 44 patients with primary right heart sarcoma, those receiving neoadjuvant chemotherapy before tumor resection showed double the median survival compared to those without (20 months versus 9.5 months) [10].

The differential diagnosis of malignant primary cardiac tumors (PCT) of the heart is broad and includes sarcomas, lymphomas, and mesotheliomas. Among the sarcoma group, angiosarcomas are the predominant histological subtype, followed by undifferentiated sarcomas [1]. In this case, the tumor was a high-grade spindle cell and pleomorphic sarcoma of the right ventricle, classified as undifferentiated due to its nonspecific histology and immunohistochemistry. Histopathological differentiation of the cardiac tumor determines the patient’s prognosis, as malignant PCT is associated with higher mortality and recurrence. Additionally, a meta-analysis found long-term mortality for malignant PCT to be 14.77% compared to 0.79% for benign cardiac tumors [5]. Oliveira et al. reported that 80% of patients with malignant PCT died within 20 months of diagnosis [11].

## Conclusions

This case highlights the importance of considering cardiac tumors in the differential diagnosis of cardiac masses. It also illustrates the poor prognosis and recurrence associated with these tumors, as well as the lack of standard protocols for their management (Additional file 1, Additional file 2, Additional file 3 and Additional file 4).

### Supplementary Information

**Additional file 1**. **Video 1** Transesophageal echocardiogram: This video shows the right ventricle cavity. There is a hypoechoic mass with regular borders that is affecting the interventricular septum and tricuspid valve.

**Additional file 2**. **Video 2** Transesophageal echocardiogram: Short-axis view of great vessels. A mass with regular borders is appreciated, inside of which hypoechoic areas that occupy the cavity of the right ventricle are evident. The mass extends at least to the level of the pulmonary valve, causing complete obliteration of the right ventricular outflow tract.

**Additional file 3**. **Video 3** Transesophageal echocardiogram: Short-axis view of great vessels. A mass with regular borders is appreciated, inside of which hypoechoic areas that occupy the cavity of the right ventricle are evident. The mass extends at least to the level of the pulmonary valve, causing complete obliteration of the right ventricular outflow tract.

**Additional file 4**. **Video 4** Transthoracic echocardiogram: Echocardiography, four-chamber view, shows a hypoechoic mass with regular borders that occupies the cavity of the right ventricle adhered to the basal interventricular septum. The lesion involves the tricuspid papillary muscle as well as affecting the tricuspid valve.

## Declarations

### Availability of data and materials

Data sharing is not applicable to this article as no datasets were generated or analyzed during the current study.

## References

[1] Tyebally S, Chen D, Bhattacharyya S, Mughrabi A, Hussain Z, Manisty C (2020). Cardiac tumors. JACC CardioOncol.

[2] Maleszewski JJ, Basso C, Bois MC, Glass C, Klarich KW, Leduc C (2022). The 2021 WHO classification of tumors of the heart. J Thorac Oncol.

[3] Butany J, Nair V, Naseemuddin A, Nair GM, Catton C, Yau T (2005). Cardiac tumours: diagnosis and management. Lancet Oncol.

[4] Antwi-Amoabeng D, Meghji Z, Thakkar S, Ulanja MB, Taha M, Adalja D (2020). Survival differences in men and women with primary malignant cardiac tumor: an analysis using the surveillance, epidemiology and end results (SEER) database from 1973 to 2015. J Am Heart Assoc.

[5] Rahouma M, Arisha MJ, Elmously A, El-Sayed Ahmed MM, Spadaccio C, Mehta K (2020). Cardiac tumors prevalence and mortality: A systematic review and meta-analysis. Int J Surg.

[6] Koo J, Knight-Perry J, Galambos C, Browne LP, Cost CR (2021). Pediatric metastatic cardiac angiosarcoma successfully treated with multimodal therapy: case report and review of literature. J Pediatr Hematol Oncol.

[7] Jain A, Simon S, Elangovan I (2015). 18F-fluoro-deoxyglucose positron emission tomography-computed tomography in initial assessment and diagnosis of right atrial angiosarcoma with widespread visceral metastases: a rare case report and review of the literature. Indian J Nucl Med IJNM Off J Soc Nucl Med India.

[8] Hasan SM, Witten J, Collier P, Tong MZ, Pettersson GB, Smedira NG (2021). Outcomes after resection of primary cardiac sarcoma. JTCVS Open.

[9] Devbhandari MP, Meraj S, Jones MT, Kadir I, Bridgewater B (2007). Primary cardiac sarcoma: reports of two cases and a review of current literature. J Cardiothorac Surg.

[10] Abu Saleh WK, Ramlawi B, Shapira OM, Al Jabbari O, Ravi V, Benjamin R (2017). Improved outcomes with the evolution of a neoadjuvant chemotherapy approach to right heart sarcoma. Ann Thorac Surg.

[11] Oliveira GH, Al-Kindi SG, Hoimes C, Park SJ (2015). Characteristics and survival of malignant cardiac tumors. Circulation.

